# Relevance of Visual Acuity Measurement for Therapeutic Decisions in Diabetic Macular Edema

**DOI:** 10.3390/pharmaceutics15061607

**Published:** 2023-05-29

**Authors:** Thibaud Mathis, Batoul El Ameen, Cristina Vartin, Yasmine Serrar, Frédéric Matonti, Aditya Sudhalkar, Alper Bilgic, Amina Rezkallah, Laurent Kodjikian

**Affiliations:** 1 Service d’Ophtalmologie, Hôpital Universitaire de la Croix-Rousse, Hospices Civils de Lyon, 69004 Lyon, France; thibaud.mathis@chu-lyon.fr (T.M.); baelameen@gmail.com (B.E.A.); christine.vartin@chu-lyon.fr (C.V.); yasmine.serrar@chu-lyon.fr (Y.S.); amina.rezkallah@chu-lyon.fr (A.R.); 2 UMR5510 MATEIS, CNRS, INSA Lyon, Université Lyon 1, 69100 Villeurbanne, France; 3 Centre Monticelli Paradis, 13008 Marseille, France; fredmatonti@gmail.com; 4 Groupe Almaviva Santé, Clinique Juge, 13008 Marseille, France; 5 MS Sudhalkar Medical Research Foundation, Baroda 390001, India; dradityasudhalkar@gmail.com; 6 Alphavision Augenarztpraxis, 27568 Bremerhaven, Germany; drbilgicalper@yahoo.com

**Keywords:** diabetic macular edema, optical coherence tomography, visual acuity

## Abstract

This study aimed to determine the validity of basing retreatment decisions on anatomical criteria alone (captured using optical coherence tomography (OCT)—OCT-guided strategy) rather than the gold standard (combined visual acuity (VA) and OCT) in patients with diabetic macular edema (DME). This cross-sectional study included 81 eyes undergoing treatment for DME from September 2021 to December 2021. An initial therapeutic treatment decision based on OCT results was made on inclusion. Subsequently, in light of the patient’s VA score, this initial decision was upheld or adjusted, and the sensitivity, specificity, positive predictive value (PPV), and negative predictive value (NPV) were calculated. In 67 out of the 81 eyes included in the study (82.7%), the OCT-guided strategy produced equivalent results to the gold standard. In this study, the OCT-guided retreatment decision strategy yielded sensitivity and specificity of 92.3% and 73.8%, respectively, and PPV and NPV of 76.6% and 91.2%, respectively. These findings differed according to the patient’s treatment regimen: the sensitivity and specificity for eyes under a treat and extend regimen was higher, 100% and 88.9%, respectively, than eyes under a Pro Re Nata regimen, 90% and 69.7%, respectively. These findings show that VA testing could be omitted from the follow-up of certain patients with DME treated with intravitreal injections without impacting the quality of care.

## 1. Introduction

For patients with diabetes mellitus, diabetic macular edema (DME) is the leading cause of visual impairment [1]. It is also associated with the upregulation of inflammatory cytokines, most notably vascular endothelial growth factor (VEGF), which alters the blood-retinal barrier leading to the extravasation of fluid into the extracellular space, which in clinical terms presents as macular edema and can lead to vision loss [2]. Currently, intravitreal injections of anti-VEGF agents, ranibizumab (Lucentis^®^, Novartis Pharma AG, Basel, Switzerland) and aflibercept (Eylea^®^, Bayer, Leverkusen, Germany), or steroids, dexamethasone implant (DEX-implant, Ozurdex^®^, AbbVie, North Chicago, IL, USA) and fluocinolone acetonide implant (FAc-implant, Iluvien^®^, Alimera Sciences Ltd., Alpharetta, GA, USA), are considered to be the standard of care for treating DME [3,4,5], and act by reducing the macular edema caused by the disease.

Optical coherence tomography (OCT) is a non-invasive, rapid imaging technique that captures in vivo images of the retina capable of revealing macular edema. Today, OCT is the most common diagnostic tool used by ophthalmologists and has had a profound impact on the diagnosis and management of retinal diseases, including DME [6]. The current gold standard for patient follow-up and retreatment decisions is the combined use of OCT image analysis and visual acuity (VA) score. However, this raises practical issues for clinicians as measuring visual acuity is time-consuming (particularly with elderly patients), and the results can vary depending on the patient’s state of fatigue (even in non-active cases).

To the best of our knowledge, while the role of OCT has been widely determined in the literature to be an essential part of retinal disease management, little attention has been paid to the role of VA scores in making retreatment decisions. It would appear that in DME patients, OCT findings provide information that can be used to establish the patient’s prognosis and follow-up without knowing their VA score [7], while some other studies failed to find an association between changes in VA and central subfield thickness on OCT in DME patients [8]. However, it remains unclear how VA scores alone can determine the need for retreatment with anti-VEGF or steroids. Since the COVID-19 pandemic, this question has become increasingly important, as ophthalmology departments were forced to change their treatment protocols by administering bilateral injections at the same time, establishing fixed treatment regimens, and, notably, canceling appointments at which patients’ VA would have been measured [9]. We recently demonstrated that certain patients with neovascular age-related macular degeneration (nAMD) under anti-VEGF treatment could be monitored without regular VA testing [10].

This study aims to establish whether retreatment decisions for patients being treated for DME could be made on the basis of anatomical criteria alone (primarily OCT-guided) instead of the current gold standard combining anatomical and functional criteria (determined based on VA score).

## 2. Materials and Methods

This cross-sectional study included patients treated for DME at the ophthalmology tertiary retinal reference center at the Croix-Rousse University Hospital in Lyon, France, between September 2021 and December 2021. The inclusion criteria were age ≥18 year old and the presence of macular edema ≥250 µm associated with diabetes mellitus treated with anti-VEGF therapy, DEX-implant, or FAc-implant at the time of inclusion. Patients were excluded if they had cataracts (causing significant visual impairment), aphakia, vitreous hemorrhage, significant vitreomacular tractional disease, a history of rhegmatogenous retinal detachment, uncontrolled glaucoma, or any other macular disease. The study complied with both the Declaration of Helsinki and French legislation currently in force. Informed consent was obtained from all the patients included, and the study was approved by a local Ethics Committee (Hospices Civils de Lyon), registered under the number 20–156.

The variables assessed in this study were VA, in a seated position using the Early Treatment Diabetic Retinopathy Study (ETDRS) scale at an initial testing distance of 4 m or at 1 m (if not possible at 4 m); OCT images obtained using a spectral-domain OCT (SD-OCT) device (HRA Spectralis, Heidelberg Engineering, Heidelberg, Germany); central retinal thickness (CRT, defined as the mean thickness of the 1 mm ETDRS grid centered on the macula); and maximal retinal thickness (MRT, defined as the maximal mean thickness found in the whole ETDRS grid). The DME was characterized as mild, moderate, or severe (depending on the distance of the thickening and exudates from the fovea) [11] using multimodal imaging including SD-OCT and fluorescein angiography (HRA Spectralis, Heidelberg Engineering, Heidelberg, Germany). A number of additional parameters were also collected: treatment regimen, duration of follow-up and treatment, the anti-VEGF molecule administered, the duration of VA measurement, the dilated fundus examination results, and the number of intravitreal injections.

The initial therapeutic decision made at the time of inclusion was based on the analysis of SD-OCT images by senior retina specialists who were not aware of the patients’ VA scores (OCT-guided strategy). This decision was then either upheld or adjusted after the specialists were made aware of the patient’s VA score in addition to SD-OCT images (OCT + VA, gold standard). For patients treated under a Pro Re Nata (PRN) regimen, the options available were to continue treatment or revert to monitoring. For patients treated under a treat and extend (TAE) regimen, the options were to continue, extend, or reduce the duration of retreatment. The retreatment decisions were discussed by two retinal specialists (B.E.A and C.V). If these two clinicians were unable to agree, a third senior specialist (L.K.) was asked to decide whether to retreat or not. All specialists were given access to the patient’s full data history (VA, OCT imaging, and retinography), as well as the retinography (Optos California system, Optos PLC, Dunfermline, Scotland, United Kingdom) and SD-OCT images obtained on the day. The VA measurements obtained on the day were then provided in a second step, and the specialist was able to modify their decision to retreat at this stage, thus establishing how the knowledge of VA scores impacted the decision made.

The decisions on the treatment regimen and retreatment were made at the ophthalmologist’s discretion, given that this was a real-life observational study. In our retinal tertiary center, retreatment was generally decided upon using international guidelines from randomized controlled trials. Furthermore, signs of active exudation (presence of SRF and/or IRF increased in CMT) on SD-OCT also usually triggered a decision to retreat, as well as a loss of ≥5 letters in visual acuity score since the patient’s last visit.

The patient data collected were anonymized and entered into an Excel spreadsheet (Microsoft Corp., Redmond, WA, USA) before processing with XLSTAT statistical software (Lumivero, Denver, CO, USA). Spearman’s test was used to perform a correlation analysis between CRT and VA scores. Sensitivity and specificity were calculated for the OCT-guided strategy compared to the gold standard of combined SD-OCT and VA. A two-by-two table was used to summarize the comparisons between the OCT-guided strategy and the gold standard. Patients for whom a decision was initially made to administer an injection (PRN regimen) or to reduce/maintain the treatment intervals (TAE regimen) based on the OCT-guided strategy and for whom this decision was confirmed after the clinician was informed of the VA score were considered true positives. Patients for whom a decision was initially made not to inject (PRN regimen) or to extend the treatment interval (TAE regimen) based on the OCT-guided strategy and subsequently confirmed after the VA score was revealed were considered true negatives. Patients for whom a decision was made to inject or to reduce/maintain the treatment intervals based on the OCT-guided strategy but were ultimately not injected after the VA score was revealed were false positives. Finally, patients for whom a decision was made not to inject or to lengthen the treatment intervals based on the OCT-guided strategy but ultimately were injected after their VA score was revealed were false negatives. The *p*-value < 0.05 was considered statistically significant.

## 3. Results

### 3.1. Patient Characteristics

This study included a total of 81 eyes in 57 patients with DME. The mean (SD) duration of follow-up from disease onset to patient inclusion was 4.6 (2.9) years; the mean (SD) number of injections was 7.0 (4.7); mean (SD) VA was 72.2 (9.8) letters at disease onset and 71.4 (17.6) letters on inclusion; mean (SD) CRT was 335.4 (101.6) µm; and the maximal retinal thickness was 380.8 (78.2) µm (Table 1).

### 3.2. Treatment Decision Changes When Informed of VA Score

In 82.7% of the cases in the study population, the retreatment decision made on the basis of anatomical criteria alone (mainly OCT-guided) was maintained when the gold standard was applied (OCT + VA score). In 17.3% of cases, the retreatment decision was adjusted when the patient’s VA score was revealed.

**Figure 1:**
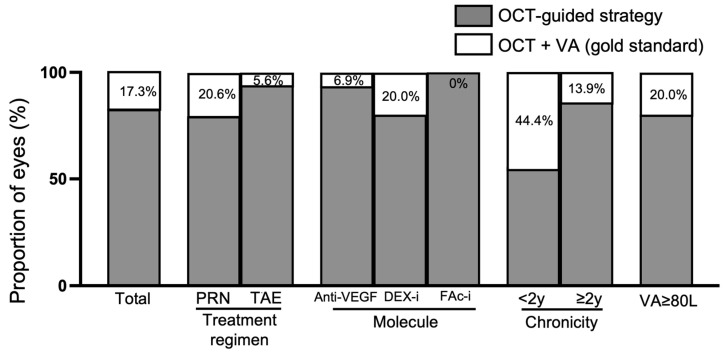
Performance of the OCT-guided strategy for retreatment decisions in comparison to the gold standard (visual acuity + OCT). DEX-i: dexamethasone implant; DME: diabetic macular edema; FAc-i: fluocinolone acetonide implant; OCT: optical coherence tomography; PRN: pro re nata; TAE: treat and extend; VA: visual acuity; anti-VEGF: anti-vascular endothelial growth factor.

There were different retreatment decision outcomes for the treatment regimen subgroups (PRN vs. TAE), as being made aware of the patient’s VA score led to a change in the decision in 20.6% of cases in the PRN subgroup and 5.6% of cases in the TAE subgroup. According to edema severity, the VA score changed the treatment decision in 4.0% of cases for mild DME, 46.1% of cases for moderate DME, and 18.9% of cases for severe DME. In terms of the treatment used, adding in the VA score changed the treatment decision in 6.9% of cases for aflibercept, 20.0% of cases for DEX-implant, and 0% of cases for FAc-implant. Regarding the chronicity of the disease, knowledge of the patient’s VA changed the treatment decision in 44.4% of cases of recent-onset DME (<2 years) in comparison to 13.9% of cases of chronic DME (≥2 years). For eyes with good vision (VA ≥ 80 letters), the information on VA changed the treatment decision in 20.0% of cases (Figure 1).

### 3.3. Sensitivity and Specificity of the OCT-Guided Strategy in Comparison to OCT and VA

In the whole population, the OCT-guided strategy had a sensitivity of 92.3% [95%CI (78.8–98.0)] and specificity of 73.8% [95%CI (58.7–84.8)]; the corresponding PPV and NPV were 76.6% [95%CI (64.5–88.7)] and 91.2% [95%CI (81.6–100)], respectively (Table 2).

Regarding the molecule used, patients under aflibercept injections showed better specificity than patients treated with DEX-implant. In terms of the treatment regimen, sensitivity and specificity were higher for eyes under a TAE regimen compared to eyes under a PRN regimen. For patients with good VA (VA ≥ 80-letters), sensitivity was 90.9% and specificity 75.0% (Table 3).

### 3.4. VA Measurement

**Figure 2:**
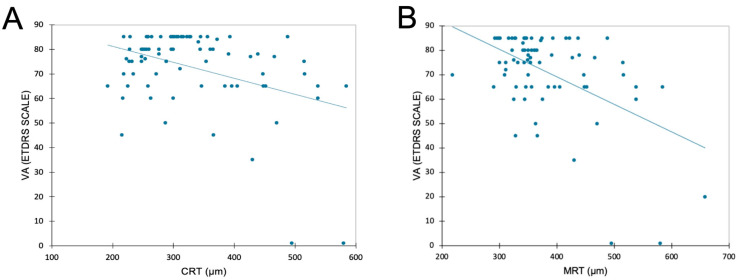
Linear regression plot for visual acuity (VA, ETDRS Scale) and central retinal thickness (CRT, panel (**A**)) or maximal retinal thickness (MRT, panel (**B**)) at the study visit. Correlation analysis performed using Spearman’s test showed a significant negative association for CRT (r = −0.39; *p* = 0.004) and MRT (r = −0.51, *p* < 0.0001).

The mean (SD) duration of visual acuity measurement was 4.8 (1.6) minutes. There was a significant but limited negative correlation between VA and CRT, with CRT increasing as VA drops (r = −0.39; *p* = 0.004, Figure 2). A negative correlation was also found between VA and MRT (r = −0.51; *p* < 0.0001).

### 3.5. Disagreement between OCT and Gold Standard

There was a total of 14 cases where the retreatment decision made based on the OCT-guided strategy did not correspond to the retreatment decision made using the gold standard (OCT + VA). Of these 14 eyes, 11 were ultimately not reinjected after taking into consideration the VA score, these were therefore considered to be false positives. In 10 out of these 11 cases, the change in the decision was explained by the presence of persistent or increased edema without VA loss; in the remaining case, it was due to late-stage DME with no functional treatment effectiveness. The other 3 out of 14 eyes were ultimately injected after taking into consideration the VA score and were therefore considered to be false negatives, a discrepancy explained by the presence of persistent or decreased DME with VA loss.

## 4. Discussion

The findings of this study show that in most cases, the decision to retreat based on anatomical criteria alone was well-informed, with sensitivity and specificity of 92.3% and 73.8%, respectively. Only 17.3% of cases saw a change in the treatment decision when the patient’s VA score was added to the anatomical criteria. However, it is true that the patients included in this study were undergoing treatment for DME at a tertiary retinal center and were not therefore expected to experience a significant change in VA between consultations. The PPV shows that the proportion of eyes with an initial OCT-guided decision to retreat that was upheld after the VA score was revealed was almost 77%. The remaining 23% (11 eyes) would have been retreated if the initial OCT-guided treatment decision had been upheld, but this decision was reversed when the VA score was revealed, and they were shown not to need treatment. The NPV shows that the proportion of eyes with an initial OCT-guided decision not to retreat that was upheld after the VA score was revealed was 91%. The remaining 9% (3 eyes) would have not received treatment if the original OCT-guided treatment decision had been upheld, but this decision was reversed when the VA score showed they did require treatment. These findings are supported by the literature, which shows that OCT can be helpful when making decisions on retreatment with anti-VEGF or steroids. However, to the best of our knowledge, there have been no studies carried out to specifically investigate the use of an OCT-guided strategy for retreatment decisions. The issue with obtaining VA scores is that it requires competent specialists and takes a long time (estimated average duration of VA measurement: 5 min per patient). Furthermore, it is well established that a given patient’s VA score can vary between two tests according to the test conditions, the conscientiousness of the VA examiner, and the patient’s level of fatigue. This is why standardized clinical studies require a threshold of at least five letters to determine significance [12]. Weak but nonetheless significant negative correlations were found between VA and CRT/MRT in this study, demonstrating that a decrease in VA is usually associated with an increase in CRT/MRT. These results show the vital contribution OCT makes to monitoring disease progression and activity by analyzing changes in the quantitative measurements it captures (CRT/MRT) and by producing a qualitative assessment of the fluid.

It is now well established that CRT and MRT can be used as quantitative features to evaluate disease activity, progression, and treatment response in DME. However, the questions of the relationship between the extent of DME and VA loss and how VA loss is preceded by anatomical changes observed on OCT are not as consensual as in nAMD [13,14]. In the latter pathology, we found better sensitivity and specificity for an OCT-guided strategy, with fewer patients that would have been incorrectly treated without the additional input of their VA score [10].

These studies demonstrate that it is not only the quantitative criteria captured on OCT images (CRT/MRT) that make an important contribution to supporting retreatment decision-making but also the qualitative criteria. Treatment decisions for DME could be guided by the presence or absence of fluid on OCT. However, there are still some situations in which the VA score is relevant when treating DME, most obviously in cases of recent DME (<2 years) in which the revelation of the patient’s VA score changed the treatment decision for more than 40% of eyes. This could be explained by the high proportion of false positives in this subgroup, probably due to the presence of fluid in the retina but excellent visual acuity for these patients with recent edema.

Interestingly, patients treated and followed under a TAE regimen showed better results than patients under a PRN regimen, probably because the anatomical changes were already the main biomarker analyzed for retreatment decisions. Indeed, the goal of a TAE regimen is to proactively prevent the loss of vision, which is why the OCT-guided decision was maintained in more than 90% of the eyes under the TAE regimen. This could explain the differences seen between molecules: aflibercept, an anti-VEGF molecule, is usually administered under a TAE regimen, whereas DEX-implants are usually administered under a PRN regimen. The case of FAc-implants is different as these eyes only had one injection in this study, explaining the absence of sensitivity and specificity for this molecule.

Analyzing our results and the descriptions of patients for whom the OCT-guided retreatment decision did not match the gold standard shows that the false positives are mostly patients with persistent or increased fluid on OCT without VA impairment. The false negatives are mostly patients with stable or decreased DME with VA loss. For these patients, different anatomical biomarkers should be investigated to ensure more accurate results.

There are a number of limitations to the present study. As a single-center study, it does not necessarily reflect DME patient management globally, although the retreatment decisions were harmonized according to the clinical scenario in question. The observational design of the study could also mean there is a certain bias in the standardization of retreatment decisions. For example, there is no consensus on retreating for persistent fluid and making this decision requires information on a number of variables, including the type of fluid, volume, and chronicity, which is a barrier to the generalization of our results across all retinal centers. In addition, both OCT imaging and VA testing are limited in resolution and accuracy, which affects the accuracy of the therapeutic decision. To the best of our knowledge, there is no significant difference between current OCT devices in terms of DME diagnosis and CRT variation. However, it is well known that VA can change between VA testing and, therefore, could modify our results [15,16].

Lastly, the cohort in this study is rather small, thus limiting our capacity to analyze specific subgroups. The study does, however, open up avenues for investigating more specific aspects in future studies. For instance, it is now well known that many factors influence the effectiveness of treatment. It could therefore be of interest to determine which test would predict long-term maintenance/improvement of visual acuity. In this case, biomarkers on OCTA have been described in AMD [17] and also for DME [18]. However, currently, we do not have guidelines that use these new features in our clinical practice. We aimed to produce a study that reflects our daily practice to answer the question of the routine utility of VA testing, especially following the COVID-19 pandemic period when VA testing was not always performed. The findings of the present study suggest that the relevance of measuring VA should be determined based on each patient’s specific case in order to reduce the length of consultations and thus improve the quality of patient care and comfort without compromising the effectiveness of treatment. These findings are particularly relevant against the backdrop of the COVID-19 pandemic, which requires measures to keep elderly patients out of hospitals and reduce their contact with the wider patient population.

## 5. Conclusions

The results of this study show that certain DME patients treated with anti-VEGF or steroids could be monitored without regular VA testing with no significant impact on performance, especially for patients under a TAE regimen. This implies that VA testing is not always required when making a decision to retreat, apart from in certain specific cases (such as recent DME < 2 years or PRN regimen), and that VA could be measured at longer intervals in order to improve the quality of care and comfort for patients by reducing the duration of consultations. Long-term studies investigating the relevance of an OCT-guided strategy as part of the overall follow-up for DME patients are needed to build on these findings.

## Figures and Tables

**Table 1:** Demographics and disease characteristics at the study visit.

Gender, *n* (%)	
Men	27 (47.4%)
Women	30 (52.6%)
Mean (SD) age, years	67.1 (10.5)
Laterality, *n* (%)	
Right	40 (49.4%)
Left	41 (50.6%)
Molecule, *n* (%)	
Aflibercept	29 (35.8%)
Dexamethasone implant	40 (49.4%)
Fluocinolone Acetonide implant	12 (14.8%)
Macular edema, *n* (%)	
Mild	25 (30.9%)
Moderate	13 (16.0%)
Severe	37 (45.7%)
Absence	6 (7.4%)
Diabetic retinopathy, *n* (%)	
Absence of DR	1 (1.2%)
mild NPDR	5 (6.2%)
moderate NPDR	17 (21.0%)
severe NPDR	13 (16.0%)
Past PPR	45 (55.6%)
Treatment regimen, *n* (%)	
PRN	63 (77.8%)
TAE	18 (22.2%)
Mean HbA1c, % (SD)	8.1 (1.3)
Mean blood pressure, mmHg (SD)	132.5 (30.6)
Mean time since first DME diagnosis, years (SD)	4.6 (2.9)
Mean time since first IVT, years (SD)	4.5 (2.9)
Mean number of injections since first diagnosis, *n* (SD)	7.0 (4.7)
Mean VA at disease onset, ETDRS letters (SD)	72.2 (9.8)
Mean VA at study visit, ETDRS letters (SD)	71.4 (17.6)
Mean CRT at study visit, µm (SD)	335.4 (101.6)

CRT: central retinal thickness; DME: diabetic macular edema; DR: diabetic retinopathy; ETDRS: early treatment diabetic retinopathy study; IVT: intravitreal injection; NPDR: non-proliferative diabetic retinopathy; PPR: panretinal photocoagulation; PRN: pro re nata; SD: standard deviation; TAE: treat and extend; VA: visual acuity.

**Table 2:** Contingency table comparing the optical coherence tomography (OCT)-guided strategy with the gold standard (OCT + visual acuity test) for decision-making on intravitreal injection (IVT) retreatment.

	IVT+ (N = 39)	IVT− (N = 42)		
OCT+ (N = 47)	36 (44.4)	11 (13.6)	⇛	Positive predictive value 76.6% (64.5–88.7)
OCT− (N = 34)	3 (3.7)	31 (38.3)	⇛	Negative predictive value 91.2% (81.6–100)
	⤋ Sensitivity 92.3% (78.8–98.0)	⤋ Specificity 73.8% (58.7–84.8)		

**Table 3:** Sensitivity and specificity of the OCT-guided strategy for retreatment decisions in DME patients.

	Sensitivity	Specificity	PPV	NPV
Whole population	92.3% [78.8–9.0]	73.8% [58.7–84.8]	76.6% [64.5–88.7]	91.2% [81.6–100]
Treatment regimen				
PRN	90.0% [73.4–97.2]	69.7% [52.5–82.7]	73.0% [58.7–87.3]	88.5% [76.2–100]
TAE	100% [65.0–100]	88.9% [54.0–99.8]	90.0% [71.4–100]	100% [100–100]
Molecule				
Aflibercept	92.9% [66.1–100]	92.3% [64.2–100]	92.9% [79.4–100]	92.3% [77.8–100]
Dexamethasone implant	92.0% [73.7–98.8]	57.1% [32.6–78.5]	79.3% [64.6–94.1]	80.0% [55.2–100]
Fluocinolone acetonide implant	-	100% [69.5–100]	-	100% [100–100]
DME severity				
Mild	100% [59.0–100]	94.4% [72.0–100]	87.5% [64.6–100]	100% [100–100]
Moderate	57.1% [25.1–84.0]	50.0% [19.0–81.0]	57.1% [20.5–93.8]	50.0% [10.0–90.0]
Severe	100% [83.9–100]	41.7% [19.4–68.1]	78.1% [63.8–92.4]	100% [100–100]
Eyes with good vision (VA ≥ 80-letters)	90.9% [59.8–100]	75.0% [54.7–88.2]	62.5% [38.8–86.2]	94.7% [84.7–100]
DME chronicity				
<2 years	80.0 [35.9–97.5]	25.0 [4.0–71.0]	57.1 [20.5–93.8]	50.0 [0–100]
≥2 years	94.1 [79.7–99.2]	78.9 [63.3–89.1]	80.0 [67.6–92.4]	93.8 [85.4–100]

DME: diabetic macular edema; NPV: negative predictive value; PPV: positive predictive value; TAE: treat and extend; PRN: Pro Re Nata; VA: visual acuity.

## Data Availability

Not applicable.

## References

[1] Klein R., Klein B.E., Moss S.E., Davis M.D., DeMets D.L. (1984). The Wisconsin Epidemiologic Study of Diabetic Retinopathy. IV. Diabetic Macular Edema. Ophthalmology.

[2] Daruich A., Matet A., Moulin A., Kowalczuk L., Nicolas M., Sellam A., Rothschild P.-R., Omri S., Gélizé E., Jonet L. (2018). Mechanisms of Macular Edema: Beyond the Surface. Prog. Retin. Eye Res..

[3] Kodjikian L., Bellocq D., Bandello F., Loewenstein A., Chakravarthy U., Koh A., Augustin A., de Smet M.D., Chhablani J., Tufail A. (2019). First-Line Treatment Algorithm and Guidelines in Center-Involving Diabetic Macular Edema. Eur. J. Ophthalmol..

[4] Kodjikian L., Bandello F., de Smet M., Dot C., Zarranz-Ventura J., Loewenstein A., Sudhalkar A., Bilgic A., Cunha-Vaz J., Dirven W. (2022). Fluocinolone Acetonide Implant in Diabetic Macular Edema: International Experts’ Panel Consensus Guidelines and Treatment Algorithm. Eur. J. Ophthalmol..

[5] Mathis T., Kodjikian L. (2022). Do New Drugs for Diabetic Macular Edema Offer a Safer Option?. Expert Opin. Drug Saf..

[6] Virgili G., Menchini F., Casazza G., Hogg R., Das R.R., Wang X., Michelessi M. (2015). Optical Coherence Tomography (OCT) for Detection of Macular Oedema in Patients with Diabetic Retinopathy. Cochrane Database Syst. Rev..

[7] Bressler S.B., Qin H., Beck R.W., Chalam K.V., Kim J.E., Melia M., Wells J.A., Diabetic Retinopathy Clinical Research Network (2012). Factors Associated with Changes in Visual Acuity and Central Subfield Thickness at 1 Year after Treatment for Diabetic Macular Edema with Ranibizumab. Arch. Ophthalmol..

[8] Bressler N.M., Odia I., Maguire M., Glassman A.R., Jampol L.M., MacCumber M.W., Shah C., Rosberger D., Sun J.K., DRCR Retina Network (2019). Association between Change in Visual Acuity and Change in Central Subfield Thickness during Treatment of Diabetic Macular Edema in Participants Randomized to Aflibercept, Bevacizumab, or Ranibizumab: A Post Hoc Analysis of the Protocol T Randomized Clinical Trial. JAMA Ophthalmol..

[9] Kodjikian L. (2020). How to approach intravitreal injections during this COVID-19 pandemic?. J. Fr. Ophtalmol..

[10] Mathis T., El Ameen B., Chaperon M., Serrar Y., Devin F., Dziadzko M., Rezkallah A., Kodjikian L. (2023). Relevance of Visual Acuity Measurement for Therapeutic Decisions in Age-Related Macular Degeneration. J. Clin. Med..

[11] Wilkinson C.P., Ferris F.L., Klein R.E., Lee P.P., Agardh C.D., Davis M., Dills D., Kampik A., Pararajasegaram R., Verdaguer J.T. (2003). Proposed International Clinical Diabetic Retinopathy and Diabetic Macular Edema Disease Severity Scales. Ophthalmology.

[12] Lovie-Kitchin J.E., Brown B. (2000). Repeatability and Intercorrelations of Standard Vision Tests as a Function of Age. Optom. Vis. Sci..

[13] Browning D.J., Glassman A.R., Aiello L.P., Beck R.W., Brown D.M., Fong D.S., Bressler N.M., Danis R.P., Kinyoun J.L., Diabetic Retinopathy Clinical Research Network (2007). Relationship between Optical Coherence Tomography–Measured Central Retinal Thickness and Visual Acuity in Diabetic Macular Edema. Ophthalmology.

[14] Schmidt-Erfurth U., Garcia-Arumi J., Bandello F., Berg K., Chakravarthy U., Gerendas B.S., Jonas J., Larsen M., Tadayoni R., Loewenstein A. (2017). Guidelines for the Management of Diabetic Macular Edema by the European Society of Retina Specialists (EURETINA). Ophthalmologica.

[15] Yu H.J., Kaiser P.K., Zamora D., Bocanegra M., Cone C., Brown D.M., Sadda S.R., Wykoff C.C. (2021). Visual Acuity Variability: Comparing Discrepancies between Snellen and ETDRS Measurements among Subjects Entering Prospective Trials. Ophthalmol. Retin..

[16] Baker C.W., Josic K., Maguire M.G., Jampol L.M., Martin D.F., Rofagha S., Sun J.K., DRCR Retina Network (2023). Comparison of Snellen Visual Acuity Measurements in Retinal Clinical Practice to Electronic ETDRS Protocol Visual Acuity Assessment. Ophthalmology.

[17] Mathis T., Dimassi S., Loria O., Sudhalkar A., Bilgic A., Denis P., Pradat P., Kodjikian L. (2021). Retinal Vascularization Analysis on Optical Coherence Tomography Angiography before and after Intraretinal or Subretinal Fluid Resorption in Exudative Age-Related Macular Degeneration: A Pilot Study. J. Clin. Med..

[18] Hsieh Y.-T., Alam M.N., Le D., Hsiao C.-C., Yang C.-H., Chao D.L., Yao X. (2019). OCT Angiography Biomarkers for Predicting Visual Outcomes after Ranibizumab Treatment for Diabetic Macular Edema. Ophthalmol. Retin..

